# Food and Nutrition Surveillance System markers predict diet quality

**DOI:** 10.11606/s1518-8787.2023057005087

**Published:** 2023-10-30

**Authors:** Maria Laura da Costa Louzada, Vanessa Del Castillo Silva Couto, Fernanda Rauber, Claudia Raulino Tramontt, Thanise Sabrina Souza Santos, Bárbara Hatzlhoffer Lourenço, Patrícia Constante Jaime

**Affiliations:** I Universidade de São Paulo Faculdade de Saúde Pública Departamento de Nutrição São Paulo SP Brazil Universidade de São Paulo . Faculdade de Saúde Pública . Departamento de Nutrição . São Paulo , SP , Brazil; II Universidade de São Paulo Faculdade de Medicina Departamento de Medicina Preventiva São Paulo SP Brazil Universidade de São Paulo . Faculdade de Medicina . Departamento de Medicina Preventiva . São Paulo , SP , Brazil; III Universidade Federal de Minas Gerais Grupo de Pesquisa de Intervenções em Nutrição Belo Horizonte MG Brazil Universidade Federal de Minas Gerais . Grupo de Pesquisa de Intervenções em Nutrição . Belo Horizonte , MG , Brazil

**Keywords:** Nutrition and Food Programs and Policies, Food and Nutrition Surveillance, Food Intake

## Abstract

**OBJECTIVE:**

To investigate the performance of food consumption markers of the Food and Nutrition Surveillance System (Sisvan) in assessing the overall dietary quality.

**METHODS:**

The study was carried out based on the reproduction of responses to markers in 24-hour recall data from 46,164 individuals aged ≥ 10 years, from the 2017–2018 Household Budget Survey (POF). Seven Sisvan markers were evaluated, and two scores were calculated for each participant, based on the sum of the number of healthy food markers (beans, fruits, and vegetables, ranging from 0 to 3) and unhealthy (hamburgers/sausages, sweetened beverages, instant noodles/salt snacks/crackers, stuffed cookies/sweets/candies, ranging from 0 to 4) consumed. Linear regression analyses were used to assess the association between scores and diet quality indicators (ultra-processed foods, dietary diversity, and levels of saturated and trans fat, added sugar, sodium, potassium, and fiber in the diet).

**RESULTS:**

The score of healthy eating markers increased significantly with increasing dietary diversity and potassium and fiber contents in the diet, while the opposite trend was observed for the densities of added sugar, sodium, saturated and trans fat (p < 0.001). The score of unhealthy eating markers increased significantly with the increase in the consumption of ultra-processed foods and densities of added sugar, saturated and trans fat levels in the diet, while an inverse trend was observed for potassium and fiber (p < 0.001). The joint analysis of the combination of the two marker scores showed that individuals with better performance (3 in the healthy food score, and 0 in the unhealthy food score) have a lower number of inadequacies in nutrient consumption.

**CONCLUSION:**

Sisvan food consumption markers, quickly and easily applied and already incorporated into the Brazilian public health system, have good potential to reflect the overall dietary quality.

## INTRODUCTION

Unhealthy eating is one of the main risk factors for mortality and disability adjusted life years, in addition to driving unprecedented environmental damage ^1^ . There is a growing consensus that a large part of this problem is related to the dramatic changes in food systems that have occurred in recent decades, mainly characterized by the rise in consumption of ultra-processed foods, together with insufficient dietary diversity ^1^ . Given this, it is a priority for national governments to routinely assess and monitor the quality of the population’s diet, with a view to identifying problems and formulating and evaluating public policies ^5^ .

Most of the dietary surveys carried out today measure information on food consumption using comprehensive data collection instruments, which demand experienced interviewers and a large amount of time, and which are more expensive, as in the case of 24-hour recalls ^6^ . However, it is essential that countries, especially low- and middle-income countries, implement surveillance systems capable of carrying out an adequate diagnosis of the population’s food intake and nutritional status using cheaper, faster, and more continuous instruments ^7^ .

In Brazil, since 1990, the Food and Nutrition Surveillance System ( *Sistema de Vigilância Alimentar e Nutricional* – Sisvan) enables the collection of data on the Brazilian population’s nutritional status and food consumption from Primary Health Care (PHC) of the Unified Health System ( *Sistema Único de Saúde* – SUS) ^10^ . Questions about food consumption were updated in 2015 so that they were aligned with the recommendations of the Dietary Guidelines for the Brazilian Population, which mainly encourages adherence to a dietary pattern that is based on a variety of fresh and minimally processed foods and restriction of ultra-processed foods ^11^ . The Sisvan form allows, based on a quick assessment of selected food consumption markers, any PHC professional to continually assess food consumption, capture healthy and unhealthy practices, and receive information to carry out guidance at all stages of the life course ^12^ . The form for people aged two years or older has nine yes/no questions regarding the previous day: two about eating habits (habit of eating in front of screens and meals eaten throughout the day), three about markers of healthy eating (consumption of beans, fresh fruits, and vegetables) and four on unhealthy eating markers (consumption of hamburgers and/or sausages, sweetened beverages, instant noodles, packaged snacks and/or crackers, and cookies and/or candies) ^13^ .

Despite being updated and implemented in the national surveillance system and in the PHC information systems, there is little evidence of evaluating these markers as a tool capable of capturing the healthiness of the diet. Thus, the objective of this study is to investigate, in the Brazilian context, the performance of Sisvan’s food consumption markers in assessing the overall dietary quality of Brazilian adolescents and adults.

## METHODS

This study was carried out from the responses to Sisvan food consumption markers in data from 24-hour recalls of a representative sample of the Brazilian population, of which the details are described below.

### Data Source and Sampling

The data analyzed make up the individual food consumption module of the *Pesquisa de Orçamentos Familiares* (POF – Household Budget Survey), carried out by the Brazilian Institute of Geography and Statistics (IBGE), between July 2017 and July 2018. A complex two-stage sampling process was carried out, with grouping of census sectors according to geographic and socioeconomic stratification and their subsequent selection in the first stage followed by the selection of households from the previously selected sectors in the second stage ^14^ .

### Data collection

Information regarding individual food consumption was collected from a subsample of 20,112 households and reported by residents aged 10 years or older. For the 46,164 individuals selected for the consumption module, 24-hour recalls on two non-consecutive days were performed. Research agents collected, in an interview consisting of several stages, information on all foods and beverages consumed on the previous day, amounts in household measures, type, preparation methods, time, occasion of consumption (breakfast, lunch, dinner, snack) and location. For some pre-selected foods (such as coffee, tea, juices, and bread), information was requested about the addition of other items, such as sugar, sweetener, olive oil, and butter/margarine. Quantities considered improbable or not informed were imputed by the IBGE based on a matrix of similarities, formed by variables considered correlated with the quantity consumed variable ^14^ .

The amount of each food or drink recorded in the recalls was transformed into grams or milliliters and converted into energy (kilocalories, kcal) and nutrients (g, mg, or µg), based on the Brazilian Food Composition Table (TBCA) of the Universidade de São Paulo (USP), Food Research Center (FoRC), Version 7.0. São Paulo, 2019. ^a^ For this study, data from the first day of the dietary survey were used.

Data referring to the interviewee’s date of birth, sex, and *per capita* family income were obtained using standardized questionnaires.

### Sisvan’s Healthy and Unhealthy Eating Marker Scores

Based on the data from the 24-hour recall, 7 variables were created corresponding to the report (yes/no) of the consumption of foods that are part of the Sisvan markers ^12^ , namely:

- Healthy eating markers:
  1. beans
  2. fruit (excluding fruit juice)
  3. vegetables (excluding potatoes, cassava, and yams)
- Unhealthy eating markers:
  1. hamburger and/or sausages (ham, *mortadela* , salami, sausage)
  2. sweetened drinks (soda, industrialized juice, powdered juice)
  3. instant noodles, prepackaged snacks, or crackers
  4. cookies, sweets, or confectionaries (candies, lollipops, gum, caramel, gelatin)

Then, a score of healthy eating markers was calculated for each individual, based on the sum of the number of healthy food groups consumed, ranging from 0 to 3, and a score of unhealthy eating markers, based on the sum of the number of unhealthy food groups consumed, ranging from 0 to 4.

To create the Sisvan scores, the different foods consumed alone or present in culinary preparations were considered, excluding vegetables potentially used as seasoning in these preparations (such as garlic, coriander, chives, parsley), or those with a secondary participation in fast food snacks (like pizza tomatoes).

### Food Quality Indicators

#### Participation of ultra-processed foods

The definition of ultra-processed foods was based on the Nova classification system and includes industrial formulations typically developed from food parts or laboratory-synthesized substances, made from numerous ingredients, such as sugars and syrups, refined starches, oils and fats, protein isolates, as well as the remains of intensively bred animals. Fresh or minimally processed foods represent reduced or zero portions in the list of ingredients of ultra-processed foods. In order to be attractive, combinations of flavorings, colorings, emulsifiers, thickeners, and other additives that modify the sensory characteristics are used. This group includes ultra-processed breads, crackers and snacks, sausages, sweets (ice cream, chocolates, candies), soft drinks, ready-to-eat or frozen meals, fast food snacks, dairy drinks, and artificial juices ^15^ . The percentage of total calories from ultra-processed foods (% of total energy) was calculated.

#### Diet diversity

Diet diversity was assessed using the Minimum Dietary Diversity indicator, proposed by the Food and Agriculture Organization of the United Nations (FAO) ^16^ . The indicator for each participant was calculated from the sum of the food groups consumed (in any amount): 1) grains, roots, and tubers 2. pulses (beans, peas, and lentils) 3. nuts and seeds 4. dairy 5. meat, poultry, and fish 6. eggs 7. dark green leafy vegetables 8. other vitamin A-rich fruits and vegetables 9. other vegetables 10. other fruits. This therefore ranges from 0 to 10.

As instructed by the FAO itself, ultra-processed items such as cookies, dairy drinks and sausages were not included in the food groups.

#### Nutrient intake

The following nutrients, which are related to the risk of obesity and several non-communicable chronic diseases ^17^ , were included in the analyses: saturated fat, trans fat, added sugar, sodium, potassium, and fiber.

Indicators related to fiber, sodium and potassium intake were expressed per 1,000 kcal, while the other nutrients were expressed as a percentage of total energy intake. In addition, using the cutoff points of the World Health Organization ^17^ , inadequate intake of added sugars (≥ 10% of total energy), saturated fats (≥ 10% of total energy), trans fats (≥ 1% of total energy), fiber (< 10 g/1000 kcal), sodium (≥ 1 g/1000 kcal), and potassium (< 1755 mg/1000 kcal) was evaluated for each individual. Additionally, an indicator was created that expresses the number of inadequacies in the consumption of nutrients, calculated from the simple sum, for each participant, of the number of nutrients with consumption outside the recommended limits (ranging, therefore, from 0 to 6).

## Data analysis

Initially, we described, from the overall population, the percentage of individuals who reported the consumption of each of the Sisvan’s healthy and unhealthy eating markers in the 24-hour survey. Next, the distributions of the scores obtained from the healthy and unhealthy eating markers were presented in graphic form.

The means of the diet quality indicators were then described, separately, according to the four categories of the healthy eating markers score (0, 1, 2, and 3) and the unhealthy eating markers score (0, 1, 2, and 3+). Linear regression analyses were used to identify the direction and statistical significance of the association between scores (exposures) and diet quality indicators (outcomes).

Finally, a joint analysis was performed based on the combination of healthy and unhealthy eating marker scores. For this, a joint variable with 16 categories was created, representing all possible combinations of scores of the four categories of the two scores (0, 1, 2, and 3 for the healthy eating markers score, and 0, 1, 2, and 3+ for the unhealthy eating markers score). The means of the number of inadequacies in nutrients consumption (which represents the sum of the number of nutrients with consumption outside the recommended limits for each participant, ranging from 0 to 6) were described according to the categories of responses combined to the healthy eating and not healthy markers. Linear regression models were used to estimate the association between the combined categories of healthy and unhealthy eating marker scores and the number of nutrient intake inadequacies, using the category with the highest healthy eating marker score (3+) and lowest unhealthy eating markers (0) (best performance in both scores) as a reference category.

Sensitivity analyses were performed by repeating all regression models with scores made by excluding, one at a time, each of the Sisvan markers. The regression models were repeated considering the adjustment for sociodemographic characteristics (age, sex, and *per capita* family income).

The calculations considered the research’s complex sampling design and its expansion factors, which make it possible to extrapolate the results to the entire Brazilian population. Analyses were performed using the Stata program, version 14 (College Station, TX: StataCorp LP).

This study complies with regulatory norms for research involving human beings in Brazil, as it uses data from a secondary source, made available by the IBGE, with guaranteed anonymity of participants.

## RESULTS

Table 1 shows the percentage of individuals who reported, in the 24-hour recalls, the consumption of each Sisvan food consumption marker. More than 50% of the participants mentioned the consumption of beans (67.6%) and vegetables (50.1%). About 1/3 mentioned the consumption of cookies, sweets, or candies (32.98%) and fruits (28.9%); and just over 1/5 the consumption of sweetened drinks (23.6%), instant noodles, packaged, snacks or crackers (21.4%), and hamburgers and/or sausages (20.8%).

**Table 1:** Percentage of individuals who report consumption in the 24-hour survey of foods considered markers of healthy and unhealthy eating by Sisvan. Brazilian population ≥ 10 years old. 2017–2018 (n = 46,164)

Food markers	% Mean (95%CI)
Healthy eating markers	
Beans	67.59 (66.36–68.80)
Fruits	28.87 (28.03–29.73)
Vegetables	50.06 (49.01–51.10)
Unhealthy eating markers	
Hamburger and/or sausages	20.76 (19.97–21.58)
Sweetened drinks	23.58 (22.74–24.45)
Instant noodles, prepackaged snacks, or crackers	21.37 (20.63–22.13)
Stuffed cookies, sweets or candies	32.98 (32.12–33.86)

Sisvan: Food and Nutrition Surveillance System; 95%CI: 95% confidence interval.

The Figure describes the distribution (in %) of the population according to the Sisvan healthy and unhealthy eating marker scores. The healthy eating markers score ranged between 0 and 3, with scores 1 and 2 being the most frequent (39.2% and 35.1%, respectively). Null and maximum scores were observed, respectively, for 13.3% and 12.4% of the population. The unhealthy eating markers score, in turn, varied between 0 and 4, with evident asymmetry to the right and concentration of values 0 and 1 (34.7% and 38.9%, respectively). Just over 1/5 of the population (29.1%) achieved a score of 2 and only 6.2% scored 3 or 4.

**Figure:**
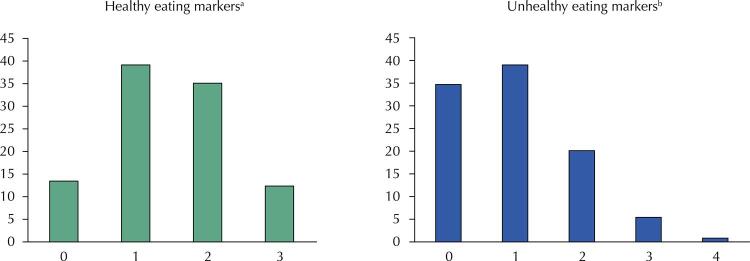
Distribution (%) of the population according to Sisvan’s scores for healthy and unhealthy eating markers c . Brazilian population ≥ 10 years old, 2017–2018 (n = 46,164).

^a^ Healthy eating markers: beans, fruits, vegetables.

^b^ Unhealthy eating markers: hamburger and/or sausages, sweetened drinks and cookies, sweets or treats.

^c^ The two scores of each participant were calculated from the sum of the number of healthy (ranging from 0 to 3) and unhealthy (ranging from 0 to 4) foods consumed.

Tables 2 and 3 show, successively, the association between Sisvan’s healthy and unhealthy eating marker scores and diet quality indicators. The score of healthy eating markers increases significantly with the increase in diversity and potassium and fiber levels in the diet, while the opposite trend is observed for the densities of added sugar, sodium, saturated and trans fat (p < 0.001). Analogously, the score of unhealthy eating markers increases significantly with the increase in the participation of ultra-processed foods and added sugar, saturated and trans fat levels in the diet, while the opposite trend is observed for potassium and fiber (p < 0.001). There was no association between the score of unhealthy eating markers and dietary sodium content.

**Table 2:** Means of diet quality indicators according to categories of healthy eating markers a,b score from Sisvan. Brazilian population ≥ 10 years old. 2017–2018 (n = 46,164)

Indicator	Healthy eating score				Coefficient	95%CI

	0	1	2	3		
Diet diversity	2.64	3.66	4.65	5.64	0.99	0.97 to 1.02 ^d^
Added sugar (% of energy)	11.79	9.89	8.98	8.61	-1.01	-1.16 to 0.83 ^d^
Sodium (g/1,000 kcal)	1.51	1.49	1.43	1.31	-0.06	-0.07 to 0.05 ^d^
Saturated fat (% of energy)	9.81	9.25	9.27	8.98	-0.2	-0.27 to 0.13 ^d^
Trans fat (% of energy)	0.79	0.7	0.64	0.59	-0.06	-0.07 to 0.05 ^d^
Potassium (mg/1,000 kcal)	992.5	1,236.40	1,398.65	1,581.71	188.34	181.26 to 195.42 ^d^
Fiber (g/1,000 kcal)	6.99	13.18	14.71	15.98	2.65	2.54 to 2.77 ^d^

Sisvan: Food and Nutrition Surveillance System; 95%CI: 95% confidence interval.

Note: Healthy food score, mean (95%CI) 1.46 (1.44–1.48).

^a^ Healthy eating markers: beans, fruits, and vegetables.

^b^ Each participant’s score was calculated from the sum of the number of markers of healthy foods consumed, ranging from 0 to 3.

^c^ Calculated from the Minimum Dietary Diversity ^16^ .

^d^ p < 0.001.

**Table 3:** Means of diet quality indicators according to categories of the score of unhealthy eating markers a,b from Sisvan. Brazilian population ≥ 10 years old, 2017–2018 (n = 46,164).

Indicator	Unhealthy eating score				Coefficient	95%CI

	0	1	two	3+		
Ultra-processed foods (% of energy)	9.66	20.02	30.06	40.24	10.2	9.74 to 10.65 ^d^
Added sugar (% of energy)	5.82	9.79	13.79	16.79	3.82	3.69 to 3.93 ^d^
Sodium (g/1,000 kcal)	1.45	1.44	1.45	1.49	0	0.00 to 0.01
Saturated fat (% of energy)	8.91	9.38	9.62	9.86	0.33	0.27 to 0.40 ^d^
Trans fat (% of energy)	0.54	0.7	0.8	0.89	0.12	0.11 to 0.13 ^d^
Potassium (mg/1,000 kcal)	1,397.11	1,312.85	1,193.51	1,081.44	-103.25	-110.65 to -95.84 ^d^
Fiber (g/1,000 kcal)	14.69	13.21	11.7	10.33	-1.47	-1.60 to -1.33 ^d^

Sisvan: Food and Nutrition Surveillance System; 95%CI: 95% confidence interval.

Note: Unhealthy food score, mean (95%CI) 0.98 (0.96–1.00).

^a^ Unhealthy eating markers: hamburger and/or sausages, sweetened beverages and cookies, sweets or treats.

^b^ The score of each participant was calculated from the sum of the number of markers of unhealthy foods consumed, ranging from 0 to 4.

^c^ Identified according to the Nova ^15^ classification .

^d^ p < 0.001.

Finally, Table 4 shows the average number of inadequacies in nutrient intake according to the 16 response categories combined with the Sisvan’s healthy and unhealthy eating markers. The highest mean number of nutrient inadequacies (5.08; SE: 0.10) is found in the group of individuals with the worst performance in both scores (0 in the healthy food score and 3+ in the unhealthy food score), while the lowest mean number of nutrient inadequacies (2.55; SE: 0.05) is found in the group of individuals with the best performance in both scores (3 in the healthy food score and 0 in the unhealthy food score). Linear regression analyses showed that the mean number of nutrient inadequacies observed in the top performers on both scores (3 on the healthy food score and 0 on the unhealthy food score) was significantly lower than the means observed in all other combined food categories (p < 0.001).

**Table 4:** Mean number of inadequacies in nutrient intake a according to ecombined exposure categories with Sisvan’s markers of healthy b and unhealthy c eating. Brazilian population ≥ 10 years old, 2017–2018 (n = 46,164).

Markers score	Unhealthy eating marker score			

	0	1	2	3+
Healthy eating marker score	Mean (EP)	Mean (EP)	Mean (EP)	Mean (EP)
0	3.93 (0.07)	4.36 (0.04)	4.74 (0.05)	5.08 (0.10)
1	3.32 (0.03)	3.80 (0.03)	4.17 (0.03)	4.72 (0.06)
2	3.11 (0.03)	3.56 (0.03)	4.02 (0.04)	4.41 (0.08)
3	2.55 (0.05)	3.12 (0.04)	3.55 (0.06)	3.95 (0.14)

Sisvan: Food and Nutrition Surveillance System; SE: standard error.

Note: The mean number of nutrient inadequacies observed in the top performers on both scores (3 on the healthy food score and 0 on the unhealthy food score) was significantly lower than the means for all other response categories (p < 0.001).

^a^ Added sugar ≥ 10% of total energy, sodium ≥ 1 g/1,000 kcal, saturated fat ≥ 10% of total energy, trans fat ≥ 1% of total energy, potassium < 1,755 mg/1,000 kcal, and fiber < 12.5g/1000kcal. ^b^ Healthy eating markers: beans, fruits, vegetables.

^c^ Unhealthy eating markers: hamburger and/or sausages, sweetened drinks and cookies, sweets or treats.

The results were not substantially modified in the sensitivity analyses, except for the association between the score of healthy eating markers and the mean saturated fat content of the diet, whose magnitude was smaller with the exclusion of beans (data not shown). Adjusting for sociodemographic variables did not significantly change the results (data not shown).

## DISCUSSION

Our results demonstrate that Sisvan food consumption markers are associated with different internationally recognized global indicators of diet quality: the participation of ultra-processed foods, diet diversity, and the content of various nutrients associated with chronic non-communicable diseases. These markers, which are part of a form applied quickly and easily in the SUS, have therefore a good potential to reflect the overall dietary quality.

Hamburgers, sausages, sweetened drinks, instant noodles, packaged snacks, cookies, and sweets, evaluated by Sisvan, are among the ultra-processed foods most consumed by the Brazilian population in 2017 and 2018 ^18^ . The participation of this food group in the diet is now considered one of the main indicators of poor diet quality, being associated with several non-communicable chronic diseases ^19^ , greater environmental footprints ^23^ , and damage to biodiversity ^24^ .

On the other hand, beans are the third fresh or minimally processed food most consumed by the Brazilian population in 2017 and 2018 ^18^ , the basis of one of the most traditional Brazilian culinary preparations and marker of consumption of a complete, healthy, and sustainable meal ^11^ . Fruits and vegetables, in turn, which are among the food groups with the highest density of vitamins and minerals, are markers of a varied diet and are consistently associated with protection against cardiovascular diseases ^25^ . Evidence of the Sisvan markers’ performance to monitor diet quality becomes even more important considering that the Brazilian population’s dietary patterns have been changing in recent decades due to the increasing replacement of culinary preparations based on fresh or minimally processed foods with ultra-processed foods ^26^ .

Nevertheless, the use of Sisvan food consumption data is presumably still not widespread in Brazilian municipalities. Considering the forms currently used in PHC, a study reported that 62.2% of Brazilian municipalities carried out at least one record of food consumption markers in 2019. In the same year, however, the population coverage of the assessment of food consumption markers was equivalent to only 0.92% of the total Brazilian population. The temporal trend of the Sisvan food consumption component in the period between 2015 and 2019 was significantly increasing, with an increase rate of 45.3% per year, which expresses substantial room for expansion of the system ^27^ .

Official documents from the Ministry of Health that guide the use of Sisvan food consumption markers highlight the possibility of their use not only to produce data for the surveillance system, but to produce care at the individual level, monitoring, and design of health promotion actions ^12^ . In this sense, Sisvan food consumption markers were recently incorporated into the Protocols for the Use of the Dietary Guidelines for the Brazilian Population in individual dietary counseling in PHC ^28^ . The protocols are official documents from the Ministry of Health that use the markers as a diagnostic tool for a person’s eating habits to guide the PHC professionals’ dietary guidelines. This choice was based on the induction of the use of markers in dietary guidance practices, in addition to the production of data for surveillance ^28^ .

Therefore, the results of this study can corroborate the strengthening of its use by presenting scientific evidence to health professionals and managers about the consistency of the information generated by the items that make up the Sisvan food consumption marker form, with potential benefits to the necessary expansion of population coverage. In addition, this new evidence can be a reference in professional qualification actions, a responsibility of SUS’s, organized under the umbrella of the National Policy on Permanent Education in Health, and carried out based on up-to-date knowledge and with the potential to impact the professional practices and orientation of the work process ^35^ .

This study has limitations arising from potential biases inherent to the relative imprecision of dietary surveys and the fact that our method did not test the application of markers via the Sisvan form in the health service. Although we are not aware of studies that have compared the indicators obtained from the Sisvan questionnaire with more comprehensive methods of collecting data on food consumption, other studies have already shown good agreement between reports of food consumption screeners and estimates from 24-hour recalls ^36 , 37^ . In addition, recent analyses, also with the objective of providing evidence of validation of Sisvan food consumption markers, indicated that the form has a two-dimensional internal structure for individuals from two years of age, opposing the four items that bring together ultra-processed foods (dimension 1) to those that include fruits, vegetables and beans (dimension 2), and has consistent measurement characteristics across macro-regions, age groups and over the years ^38^ .

On the other hand, major strengths of the study include the rigorously probabilistic character of the studied sample, and the national representativeness, ensured with the study of more than 40 thousand people residing in urban and rural areas of the various regions of the country, the collection of food consumption data carried out with different quality control strategies and through validated software, in addition to having a database with more than 2000 food items. Furthermore, the consistency of the findings can be observed with the evaluation of Sisvan markers in comparison with different widely recognized indicators of diet quality.

In conclusion, this study demonstrated that Sisvan food consumption markers perform well in reflecting the overall dietary quality in Brazilian adolescents and adults. Considering that these markers are incorporated into the SUS and are quickly and easily applied by PHC professionals, their central role in the country’s food and nutrition surveillance strategy is reaffirmed.

## Footnotes

a Available from: http://www.fcf.usp.br/tbca
