# Supplementary material for: Food and Nutrition Surveillance System markers predict diet quality
Source: Rev Saude Publica. 2023 Oct 30;57:82. doi: 10.11606/s1518-8787.2023057005087 (PMC10631750; doi:10.11606/s1518-8787.2023057005087)
Supplement: Translation

# Translation: Marcadores do Sistema de Vigilância Alimentar e Nutricional predizem qualidade da dieta

**Authors:** Maria Laura da Costa Louzada, Vanessa Del Castillo Silva Couto, Fernanda Rauber, Claudia Raulino Tramontt, Thanise Sabrina Souza Santos, Bárbara Hatzlhoffer Lourenço, Patrícia Constante Jaime

**Affiliations:** I Brazil Universidade de São Paulo. Faculdade de Saúde Pública. Departamento de Nutrição. São Paulo, SP, Brazil; II Brazil Universidade de São Paulo. Faculdade de Medicina. Departamento de Medicina Preventiva. São Paulo, SP, Brazil; III Brazil Universidade Federal de Minas Gerais. Grupo de Pesquisa de Intervenções em Nutrição. Belo Horizonte, MG, Brazil

**Keywords:** Programas e Políticas de Nutrição e Alimentação, Vigilância Alimentar e Nutricional, Ingestão de Alimentos

## Abstract

**OBJETIVO:**

Investigar o desempenho dos marcadores do consumo alimentar do Sistema de Vigilância Alimentar e Nutricional (Sisvan) na avaliação da qualidade global da alimentação.

**MÉTODOS:**

O estudo foi realizado a partir da reprodução de respostas aos marcadores em dados de recordatórios de 24 horas, de 46.164 indivíduos com idade menor ou igual a 10 anos, da Pesquisa de Orçamentos Familiares 2017–2018. Foram avaliados sete marcadores do Sisvan e calculados dois escores para cada participante, a partir do somatório do número de marcadores de alimentação saudável (feijão, frutas, verduras/legumes, variando de 0 a 3) e não saudável (hambúrguer/embutidos, bebidas adoçadas, macarrão instantâneo/salgadinhos/biscoitos salgados, biscoito recheado/doces/guloseimas, variando de 0 a 4) consumidos. Análises de regressão linear foram usadas para avaliar a associação entre os escores e indicadores de qualidade da alimentação (participação de alimentos ultraprocessados, diversidade e teores de gordura saturada, trans, açúcar de adição, sódio, potássio e fibra da dieta).

**RESULTADOS:**

o escore de marcadores de alimentação saudável aumentou de forma significativa com o aumento da diversidade e dos teores de potássio e fibra da dieta, enquanto tendência oposta foi observada para as densidades de açúcar de adição, sódio, gordura saturada e trans (p < 0,001). Observou-se que o escore de marcadores de alimentação não saudável aumentou de forma significativa com o aumento da participação de alimentos ultraprocessados e dos teores de açúcar de adição, gordura saturada e trans da dieta, enquanto tendência inversa é observada para potássio e fibra (p < 0,001). A análise conjunta da combinação dos dois escores de marcadores mostrou que indivíduos com melhor desempenho (3 no escore de alimentos saudáveis, e 0 no de alimentos não saudáveis) possuem menor número de inadequações no consumo de nutrientes.

**CONCLUSÃO:**

Os marcadores do consumo alimentar do Sisvan, aplicados de forma rápida e prática e já incorporados no sistema público de saúde brasileiro, possuem bom potencial para refletir a qualidade global da alimentação.

## INTRODUÇÃO

A alimentação não saudável é um dos principais fatores de risco para mortalidade e perda de anos de vida por incapacidade, além de impulsionar danos ambientais sem precedentes ^1^ . É cada vez maior o consenso de que grande parte desse problema está relacionado às mudanças dramáticas nos sistemas alimentares ocorridas nas últimas décadas, caracterizadas, principalmente, pela ascensão do consumo de alimentos ultraprocessados, juntamente com a insuficiente diversidade da dieta ^1^ . Diante disso, é prioridade dos governos nacionais a avaliação rotineira e o monitoramento da qualidade da alimentação da população, com vistas à identificação de problemas, formulação e avaliação de políticas públicas ^5^ .

Grande parte dos inquéritos dietéticos realizados hoje mensura informações sobre consumo alimentar pelo emprego de instrumentos de coleta de dados abrangentes, que demandam entrevistadores experientes e grande quantidade de tempo, e que possuem maior custo, como no caso dos recordatórios alimentares de 24 horas ^6^ . Entretanto, é fundamental que os países, principalmente de baixa e média renda, implementem sistemas de vigilância capazes de realizar diagnóstico adequado da situação alimentar e nutricional da população a partir de instrumentos mais baratos, rápidos e contínuos ^7^ .

No Brasil, desde 1990, o Sistema de Vigilância Alimentar e Nutricional (Sisvan) viabiliza a coleta de dados sobre o estado nutricional e o consumo alimentar da população brasileira a partir da Atenção Primária à Saúde (APS) do Sistema Único de Saúde (SUS) ^10^ . As questões sobre consumo alimentar foram atualizadas em 2015 para que estivessem alinhadas com as recomendações do Guia Alimentar para a População Brasileira, que estimula principalmente a adesão de um padrão alimentar que tem como base uma diversidade de alimentos *in natura* e minimamente processados e a restrição de alimentos ultraprocessados ^11^ . O formulário do Sisvan permite que, a partir de uma avaliação rápida de marcadores do consumo de alimentos selecionados, seja possível que qualquer profissional de saúde da APS possa realizar continuamente a avaliação do consumo alimentar, consiga captar as práticas saudáveis e não saudáveis e tenha subsídios para realizar orientações em todos as fases do curso da vida ^12^ . O formulário para pessoas com dois anos ou mais possui nove questões do tipo sim/não em relação ao dia anterior: duas sobre modos de comer (hábito de realizar refeições em frente às telas e refeições realizadas ao longo do dia), três sobre marcadores de alimentação saudável (consumo de feijão, frutas frescas e verduras e/ou legumes) e quatro sobre marcadores de alimentação não saudável (consumo de hambúrguer e/ou embutidos, bebidas adoçadas, macarrão instantâneo, salgadinho de pacote e/ou biscoitos salgados e biscoito recheado e/ou guloseimas) ^13^ .

Apesar da atualização e de estar implementado no sistema nacional de vigilância e nos sistemas de informação da APS, há escassez de evidências de avaliação destes marcadores como uma ferramenta capaz de captar a saudabilidade da alimentação. Desta forma, o objetivo deste estudo é investigar, no contexto brasileiro, o desempenho dos marcadores do consumo alimentar do Sisvan na avaliação da qualidade global da alimentação de adolescentes e adultos brasileiros.

## MÉTODOS

Este estudo foi realizado com a reprodução de respostas aos marcadores do consumo alimentar do Sisvan em dados de recordatórios de 24 horas de uma amostra representativa da população brasileira, cujos detalhes estão descritos a seguir.

### Fonte de Dados e Amostragem

Os dados analisados compõem o módulo de consumo alimentar pessoal da Pesquisa de Orçamentos Familiares (POF), realizada pelo Instituto Brasileiro de Geografia e Estatística (IBGE), entre julho de 2017 e julho de 2018. Um processo de amostragem complexa em dois estágios foi realizado, com agrupamento de setores censitários com estratificação geográfica e socioeconômica, e posterior sorteio deles no primeiro estágio, além de sorteio de domicílios pertencentes aos setores selecionados no segundo estágio ^14^ .

### Coleta de Dados

As informações referentes ao consumo alimentar individual foram coletadas em uma subamostra de 20.112 domicílios e reportadas por moradores com 10 anos de idade ou mais. Aos 46.164 indivíduos selecionados para o módulo de consumo, foram aplicados recordatórios de 24 horas em dois dias não consecutivos. Agentes de pesquisa coletaram, em entrevista composta por diversos estágios, informações sobre todos os alimentos e bebidas consumidos no dia anterior, quantidades em medidas caseiras, tipo, métodos de preparação, horário, ocasião de consumo (café da manhã, almoço, jantar, lanche) e local. Para alguns alimentos pré-selecionados (como café, chá, sucos e pães), solicitaram-se informações sobre adição de outros itens, como açúcar, adoçante, azeite e manteiga/margarina. Quantidades consideradas improváveis ou não informadas foram imputadas pelo IBGE com base em matriz de similaridades, formadas por variáveis consideradas correlacionadas com a variável quantidade consumida ^14^ .

A quantidade de cada alimento ou bebida registrada nos recordatórios foi transformada em gramas ou mililitros e convertidas em energia (quilocalorias, kcal) e nutrientes (g, mg ou µg), com base na Tabela Brasileira de Composição de Alimentos (TBCA) da Universidade de São Paulo (USP), Food Research Center (FoRC), Versão 7.0. São Paulo, 2019. ^a^ Para este estudo foram utilizados os dados do primeiro dia do inquérito alimentar.

Os dados referentes a data de nascimento, sexo e renda familiar *per capita* do entrevistado foram obtidos utilizando-se questionários padronizados.

### Escores de Marcadores de Alimentação Saudável e Não Saudável do Sisvan

A partir dos dados do recordatório de 24 horas, foram criadas 7 variáveis correspondentes ao relato (sim/não) do consumo dos alimentos que fazem parte dos marcadores do Sisvan ^12^ . São elas:

- Marcadores de alimentação saudável:
  1. feijão;
  2. frutas frescas (excluindo-se suco de frutas);
  3. verduras e/ou legumes (excluindo-se batata, mandioca, aipim, macaxeira, cará e inhame).
- Marcadores de alimentação não saudável:
  1. hambúrguer e/ou embutidos (presunto, mortadela, salame, linguiça, salsicha e similares);
  2. bebidas adoçadas (refrigerante, suco de caixinha, suco em pó e similares);
  3. macarrão instantâneo, salgadinhos de pacote ou biscoitos salgados;
  4. biscoito recheado, doces ou guloseimas (balas, pirulitos, chiclete, caramelo, gelatina e similares).

Em seguida, foi calculado, para cada indivíduo, um escore de marcadores de alimentação saudável, a partir do somatório do número de grupos de alimentos saudáveis consumidos, variando de 0 a 3, e um escore de marcadores de alimentação não saudável, a partir do somatório do número de grupos de alimentos não saudáveis consumidos, variando de 0 a 4.

Para a criação dos escores do Sisvan, foram considerados os diferentes alimentos consumidos isoladamente ou presentes em preparações culinárias, excluindo-se verduras e legumes potencialmente usados como temperos nessas preparações (como alho, coentro, cebolinha, salsinha), ou aqueles com participação secundária em lanches do tipo *fast food* (como o tomate da pizza).

### Indicadores de Qualidade da Alimentação

#### Participação de alimentos ultraprocessados

A definição de alimentos ultraprocessados foi baseada no sistema de classificação Nova e inclui formulações industriais tipicamente desenvolvidas a partir de partes de alimentos ou de substâncias sintetizadas em laboratório, feitas de inúmeros ingredientes, como açúcares e xaropes, amidos refinados, óleos e gorduras, isolados proteicos, além de restos de animais de criação intensiva. Alimentos *in natura* ou minimamente processados representam porções reduzidas ou nulas na lista de ingredientes dos alimentos ultraprocessados. Com o objetivo de serem atrativos, utilizam-se combinações de flavorizantes, corantes, emulsificantes, espessantes e outros aditivos que modificam as características sensoriais. Nesse grupo estão pães ultraprocessados, bolachas e salgadinhos, embutidos, guloseimas (sorvetes, chocolates, balas), refrigerantes, refeições prontas para o consumo ou congeladas, lanches do tipo *fast food* , bebidas lácteas e sucos artificiais ^15^ . Foi calculado o percentual do total de calorias proveniente de alimentos ultraprocessados (% do total de energia).

#### Diversidade da dieta

A diversidade da dieta foi avaliada por meio do indicador Diversidade Alimentar Mínima, proposto pela Organização das Nações Unidas para Agricultura e Alimentação (FAO) ^16^ . O indicador para cada participante foi calculado a partir do somatório dos grupos de alimentos consumidos (em qualquer quantidade): 1) cereais, raízes e tubérculos; 2) leguminosas; 3) nozes e sementes; 4) lácteos; 5) carnes, aves e peixes; 6) ovos; 7) vegetais folhosos verde-escuros; 8) outras frutas e vegetais ricos em vitamina A; 9) outros vegetais; (10) outras frutas. Este varia, portanto, de 0 a 10.

Tal como instruído pela própria FAO, não foram computados nos grupos de alimentos itens ultraprocessados tais como bolachas, bebidas lácteas e embutidos.

#### Ingestão de nutrientes

Os seguintes nutrientes, relacionados ao risco de obesidade e diversas doenças crônicas não transmissíveis ^17^ , foram incluídos nas análises: gordura saturada, gordura trans, açúcar de adição, sódio, potássio e fibra.

Os indicadores relativos à ingestão de fibras, sódio e potássio foram expressos por 1.000 kcal, enquanto os demais nutrientes foram expressos em porcentagem da ingestão total de energia. Além disso, usando os pontos de corte da Organização Mundial da Saúde ^17^ , foi avaliada, para cada indivíduo, a ingestão inadequada de açúcares de adição (≥ 10% do total de energia), gorduras saturadas (≥ 10% do total de energia), gorduras trans (≥ 1% do total de energia), fibra (< 10 g/1000 kcal), sódio (≥ 1 g/1000 kcal) e potássio (< 1755 mg/1000 kcal). Adicionalmente, foi criado um indicador que expressa o número de inadequações no consumo de nutrientes, feito a partir da simples soma, para cada participante, do número de nutrientes com consumo fora dos limites recomendados (variando, portanto, de zero a seis).

## Análise de Dados

Inicialmente, descrevemos para o conjunto da população o percentual de indivíduos que relataram o consumo de cada um dos marcadores de alimentação saudável e não saudável do Sisvan no inquérito de 24 horas. A seguir, as distribuições dos escores obtidos a partir dos marcadores de alimentação saudável e não saudável foram apresentadas na forma gráfica.

As médias dos indicadores de qualidade da alimentação foram então descritas, separadamente, segundo as quatro categorias do escore de marcadores de alimentação saudável (0, 1, 2 e 3) e do escore de marcadores de alimentação não saudável (0, 1, 2 e 3+). Análises de regressão linear foram empregadas para identificação da direção e do significado estatístico da associação entre os escores (exposições) e os indicadores de qualidade da alimentação (desfechos).

Por fim, foi realizada uma análise conjunta a partir da combinação dos escores de marcadores de alimentação saudável e não saudável. Para isso, foi criada uma variável conjunta com 16 categorias, representando todas as combinações possíveis de pontuação das quatro categorias dos dois escores (0, 1, 2 e 3 para o escore de marcadores de alimentação saudável, e 0, 1, 2 e 3+ para o escore de marcadores de alimentação não saudável). Foram descritas as médias do número de inadequações no consumo de nutrientes (que representa a soma do número de nutrientes com consumo fora dos limites recomendados para cada participante, variando de 0 a 6) de acordo com as categorias de respostas combinadas aos marcadores de alimentação saudável e não saudável. Modelos de regressão linear foram usados para estimar a associação entre as categorias combinadas dos escores de marcadores de alimentação saudável e não saudável e o número de inadequações no consumo de nutrientes, usando a categoria com o escore mais alto de marcadores de alimentação saudável (3+) e mais baixa de marcadores de alimentação não saudável (0) (melhor desempenho nos dois escores) como categoria de referência.

Análises de sensibilidade foram realizadas repetindo-se todos os modelos de regressão com escores feitos com a exclusão, um por vez, de cada um dos marcadores do Sisvan. Os modelos de regressão foram repetidos considerando o ajuste para características sociodemográficas (idade, sexo e renda familiar *per capita* ).

Os cálculos levaram em conta o delineamento amostral complexo da pesquisa e seus fatores de expansão, que possibilitam a extrapolação dos resultados para toda a população brasileira. As análises foram realizadas no programa Stata, versão 14 (College Station, TX: StataCorp LP).

Este estudo está em conformidade com as normas regulamentadoras de pesquisas envolvendo seres humanos no Brasil, pois utiliza dados de fonte secundária, disponibilizados pelo IBGE, com garantia de anonimato dos participantes.

## RESULTADOS

A Tabela 1 apresenta o percentual de indivíduos que relataram, no inquérito de 24 horas, o consumo de cada marcador do consumo alimentar do Sisvan. Mais de 50% dos participantes referiram o consumo de feijão (67,6%) e verduras e/ou legumes (50,1%). Cerca de 1/3 referiu o consumo de biscoito recheado, doces ou guloseimas (32,98%) e frutas (28,9%) e pouco mais de 1/5 o consumo de bebidas adoçadas (23,6%), macarrão instantâneo, salgadinhos de pacote ou biscoitos salgados (21,4%) e hambúrguer e/ou embutidos (20,8%).

**Tabela 1:** Percentual de indivíduos que relatam o consumo, no inquérito de 24 horas, de alimentos considerados marcadores de alimentação saudável e não saudável do Sisvan. População brasileira ≥ 10 anos de idade. 2017–2018 (n = 46.164)

Variável	% Média (IC95%)
Marcadores de alimentação saudável	
Feijão	67,59 (66,36–68,80)
Frutas	28,87 (28,03–29,73)
Verduras e/ou legumes	50,06 (49,01–51,10)
Marcadores de alimentação não saudável	
Hambúrguer e/ou embutidos	20,76 (19,97–21,58)
Bebidas adoçadas	23,58 (22,74–24,45)
Macarrão instantâneo, salgadinhos de pacote ou biscoitos salgados	21,37 (20,63–22,13)
Biscoito recheado, doces ou guloseimas	32,98 (32,12–33,86)

Sisvan: Sistema de Vigilância Alimentar e Nutricional; IC95%: intervalo de confiança de 95%.

A Figura descreve a distribuição (em %) da população segundo os escores de marcadores de alimentação saudável e não saudável do Sisvan. O escore de marcadores de alimentação saudável variou entre 0 e 3, sendo os mais frequentes os escores 1 e 2 (39,2% e 35,1%, respectivamente). Os escores nulo e máximo foram observados, respectivamente, para 13,3% e 12,4% da população. O escore de marcadores de alimentação não saudável, por sua vez, variou entre 0 e 4, com evidente assimetria à direita e concentração de valores 0 e 1 (34,7% e 38,9%, respectivamente). Pouco mais de 1/5 da população (29,1%) alcançou escore 2 e somente 6,2% escores de 3 ou 4.

**Figura:** Distribuição (%) da população segundo os escores de marcadores de alimentação saudável e não saudável c do Sisvan. População brasileira ≥ 10 anos de idade, 2017–2018 (n = 46.164).

^a^ Marcadores de alimentação saudável: feijão, frutas, verduras e/ou legumes.

^b^ Marcadores de alimentação não saudável: hambúrguer e/ou embutidos, bebidas adoçadas e biscoito recheado, doces ou guloseimas.

^c^ Os dois escores de cada participante foram calculados a partir da somatória do número dos alimentos saudáveis (variado de 0 a 3) e não saudáveis (variando de 0 a 4) consumidos.

As Tabelas 2 e 3 apresentam, sucessivamente, a associação entre os escores de marcadores de alimentação saudável e não saudável do Sisvan e indicadores de qualidade da alimentação. Observa-se que o escore de marcadores de alimentação saudável aumenta de forma significativa com a ampliação da diversidade e dos teores de potássio e fibra da dieta, enquanto tendência oposta é observada para as densidades de açúcar de adição, sódio, gordura saturada e trans (p < 0,001). De forma análoga, observa-se que o escore de marcadores de alimentação não saudável aumenta de forma significativa com a ampliação da participação de alimentos ultraprocessados e dos teores de açúcar de adição, gordura saturada e trans da dieta, enquanto tendência inversa é observada para potássio e fibra (p < 0,001). Não houve associação entre o escore de marcadores de alimentação não saudável e o teor de sódio da dieta.

**Tabela 2:** Médias de indicadores de qualidade da alimentação segundo categorias do escore de marcadores de alimentação saudável a,b do Sisvan. População brasileira ≥ 10 anos de idade. 2017–2018 (n = 46.164)

Indicador	Escore de alimentação saudável				Coeficiente	IC95%

	0	1	2	3		
Diversidade da dieta	2,64	3,66	4,65	5,64	0,99	0,97 a 1,02 ^d^
Açúcar de adição (% da de energia)	11,79	9,89	8,98	8,61	-1,01	-1,16 a 0,83 ^d^
Sódio (g/1.000 kcal)	1,51	1,49	1,43	1,31	-0,06	-0,07 a 0,05 ^d^
Gordura saturada (% da energia)	9,81	9,25	9,27	8,98	-0,2	-0,27 a 0,13 ^d^
Gordura trans (% da energia)	0,79	0,7	0,64	0,59	-0,06	-0,07 a 0,05 ^d^
Potássio (mg/1.000 kcal)	992,5	1.236,40	1.398,65	1.581,71	188,34	181,26 a 195,42 ^d^
Fibra (g/1.000 kcal)	6,99	13,18	14,71	15,98	2,65	2,54 a 2,77 ^d^

Sisvan: Sistema de Vigilância Alimentar e Nutricional; IC95%: intervalo de confiança de 95%.

Nota: Escore alimentos saudáveis, média (IC95%) 1,46 (1,44–1,48).

^a^ Marcadores de alimentação saudável: feijão, frutas e verduras e/ou legumes.

^b^ O escore de cada participante foi calculado a partir da somatória do número de marcadores de alimentos saudáveis consumidos, variando de 0 a 3.

^c^ Calculada a partir do Minimum Dietary Diversity ^16^ .

^d^ p < 0,001.

**Tabela 3:** Médias de indicadores de qualidade da alimentação segundo categorias do escore de marcadores de alimentação não saudável a,b do Sisvan. População brasileira ≥ 10 anos de idade, 2017–2018 (n = 46.164).

Indicador	Escore de alimentação não saudável				Coeficiente	IC95%

	0	1	2	3+		
Alimentos ultraprocessados (% da energia)	9,66	20,02	30,06	40,24	10,2	9,74 a 10,65 ^d^
Açúcar de adição (% da energia)	5,82	9,79	13,79	16,79	3,82	3,69 a 3,93 ^d^
Sódio (g/1.000 kcal)	1,45	1,44	1,45	1,49	0	0,00 a 0,01
Gordura saturada (% da energia)	8,91	9,38	9,62	9,86	0,33	0,27 a 0,40 ^d^
Gordura trans (% da energia)	0,54	0,7	0,8	0,89	0,12	0,11 a 0,13 ^d^
Potássio (mg/1.000 kcal)	1.397,11	1.312,85	1.193,51	1.081,44	-103,25	-110,65 a -95,84 ^d^
Fibra (g/1.000 kcal)	14,69	13,21	11,7	10,33	-1,47	-1,60 a -1,33 ^d^

Sisvan: Sistema de Vigilância Alimentar e Nutricional; IC95%: intervalo de confiança de 95%.

Nota: Escore alimentos não saudáveis, média (IC95%) 0,98 (0,96–1,00).

^a^ Marcadores de alimentação não saudável: hambúrguer e/ou embutidos, bebidas adoçadas e biscoito recheado, doces ou guloseimas.

^b^ O escore de cada participante foi calculado a partir da somatória do número de marcadores de alimentos não saudáveis consumidos, variando de 0 a 4.

^c^ Identificado segundo a classificação Nova ^15^ .

^d^ p < 0,001.

Por fim, a Tabela 4 mostra a média do número de inadequações no consumo de nutrientes de acordo com as 16 categorias de respostas combinadas aos marcadores de alimentação saudável e não saudável do Sisvan. Observa-se que o maior número médio de inadequações de nutrientes (5,08; EP: 0,10) encontra-se no grupo de indivíduos com pior desempenho nos dois escores (0 no escore de alimentos saudáveis e 3+ no escore de alimentos não saudáveis), enquanto o menor número médio de inadequações de nutrientes (2,55; EP: 0,05) encontra-se no grupo de indivíduos com melhor desempenho nos dois escores (3 no escore de alimentos saudáveis e 0 no escore de alimentos não saudáveis). Análises de regressão linear mostraram que o número médio de inadequações de nutrientes observado nos indivíduos de melhor desempenho nos dois escores (3 no escore de alimentos saudáveis e 0 no escore de alimentos não saudáveis) foi significativamente inferior às médias observadas em todas as outras categorias de respostas combinadas (p < 0,001).

**Tabela 4:** Média do número de inadequações no consumo de nutrientes a de acordo com as categorias de exposições combinadas aos marcadores de alimentação saudável b e não saudável c do Sisvan. População brasileira ≥ 10 anos de idade, 2017–2018 (n = 46.164).

Variável	Escore de marcadores de alimentação não saudável			

	0	1	2	3+
Escore de marcadores de alimentação saudável	Média (EP)	Média (EP)	Média (EP)	Média (EP)
0	3,93 (0,07)	4,36 (0,04)	4,74 (0,05)	5,08 (0,10)
1	3,32 (0,03)	3,80 (0,03)	4,17 (0,03)	4,72 (0,06)
2	3,11 (0,03)	3,56 (0,03)	4,02 (0,04)	4,41 (0,08)
3	2,55 (0,05)	3,12 (0,04)	3,55 (0,06)	3,95 (0,14)

Sisvan: Sistema de Vigilância Alimentar e Nutricional; EP: erro-padrão.

Nota: O número médio de inadequações de nutrientes observado nos indivíduos de melhor desempenho nos dois escores (3 no escore de alimentos saudáveis e 0 no escore de alimentos não saudáveis) foi significativamente inferior às médias observadas em todas as outras categorias de respostas (p < 0,001).

^a^ Açúcar de adição ≥ 10% do total de energia, sódio ≥ 1 g/1.000 kcal, gordura saturada ≥ 10% do total de energia, gordura trans ≥ 1% do total de energia, potássio < 1.755 mg/1.000 kcal e fibra < 12.5 g/1.000 kcal.

^b^ Marcadores de alimentação saudável: feijão, frutas, verduras e/ou legumes.

^c^ Marcadores de alimentação não saudável: hambúrguer e/ou embutidos, bebidas adoçadas e biscoito recheado, doces ou guloseimas.

Os resultados não foram substancialmente modificados nas análises de sensibilidade, com exceção da associação entre o escore de marcadores de alimentação saudável e o teor médio de gordura saturada da dieta, cuja magnitude ficou menor com a exclusão do feijão (dados não apresentados). O ajuste para variáveis sociodemográficas não modificou os resultados de forma relevante (dados não apresentados).

## DISCUSSÃO

Nossos resultados demonstram que os marcadores do consumo alimentar do Sisvan estão associados a diferentes indicadores globais de qualidade da dieta, reconhecidos internacionalmente: a participação de alimentos ultraprocessados, a diversidade da dieta e o teor de diversos nutrientes associados a doenças crônicas não transmissíveis. Esses marcadores, que fazem parte de um formulário aplicado de forma rápida e prática no SUS, possuem, portanto, bom potencial para refletirem a qualidade global da alimentação.

Hambúrguer, embutidos, bebidas adoçadas, macarrão instantâneo, salgadinho de pacote, biscoitos e guloseimas, avaliados pelo Sisvan, estão entre os alimentos ultraprocessados mais consumidos pela população brasileira em 2017 e 2018 ^18^ . A participação desse grupo de alimentos na dieta é hoje considerada um dos principais indicadores de má qualidade da alimentação, estando associada a diversas doenças crônicas não transmissíveis ^19^ , maiores pegadas ambientais ^23^ e prejuízos à biodiversidade ^24^ .

Por outro lado, o feijão é o terceiro alimento *in natura* ou minimamente processado mais consumido pela população brasileira em 2017 e 2018 ^18^ , base de uma das mais tradicionais preparações culinárias brasileiras e marcador do consumo de uma refeição completa, saudável e sustentável ^11^ . Frutas, verduras e legumes, por sua vez, que estão entre os grupos de alimentos com maior densidade de vitaminas e minerais, são marcadores de uma dieta variada e estão consistentemente associados à proteção para doenças cardiovasculares ^25^ . Evidências do desempenho dos marcadores do Sisvan para monitorar a qualidade da alimentação tornam-se ainda mais importantes considerando que os padrões alimentares da população brasileira vêm se modificando nas últimas décadas pela crescente substituição das preparações culinárias baseadas em alimentos *in natura* , ou minimamente processados, por alimentos ultraprocessados ^26^ .

Apesar disso, é presumível que o uso dos dados de consumo alimentar do Sisvan ainda seja pouco capilarizado nos municípios brasileiros. Considerando os formulários atualmente empregados na APS, um estudo descreveu que 62,2% dos municípios brasileiros realizaram pelo menos um registro de marcadores do consumo alimentar em 2019. No mesmo ano, contudo, a cobertura populacional da avaliação dos marcadores do consumo alimentar equivaleu a apenas 0,92% do total da população brasileira. A tendência temporal do componente de consumo alimentar do Sisvan no período entre 2015 e 2019 foi significativamente crescente, com taxa de incremento de 45,3% ao ano, o que expressa substancial espaço para expansão do sistema ^27^ .

Os documentos oficiais do Ministério da Saúde que orientam o uso dos marcadores do consumo alimentar do Sisvan destacam a possibilidade da sua utilização não só para produção de dados para o sistema de vigilância, mas para a produção do cuidado no nível individual, acompanhamento e elaboração de ações de promoção à saúde ^12^ . Nesse sentido, os marcadores do consumo alimentar do Sisvan foram recentemente incorporados aos Protocolos de Uso do Guia Alimentar para a População na orientação alimentar individual na APS ^28^ . Os protocolos são documentos oficiais do Ministério da Saúde que utilizam os marcadores como o instrumento de diagnóstico das práticas alimentares da pessoa atendida para guiar as orientações alimentares de profissionais de saúde da APS. Essa escolha teve como fundamento a indução do uso dos marcadores nas práticas de orientação alimentar, para além da produção de dados para a vigilância ^28^ .

Logo, os resultados deste estudo podem corroborar para o fortalecimento do seu uso ao apresentar evidências científicas para os profissionais de saúde e gestores sobre a consistência da informação gerada pelos itens que compõem o formulário de marcadores do consumo alimentar do Sisvan, com potenciais benefícios à necessária evolução da cobertura populacional. Além disso, essa nova evidência pode ser referência em ações de qualificação profissional, que são de responsabilidade do SUS, organizadas sob o guarda-chuva da Política Nacional de Educação Permanente em Saúde, e realizadas com base em conhecimentos atualizados e com potencial de impactar as práticas profissionais e a orientação do processo de trabalho ^35^ .

Este estudo apresenta limitações oriundas de vieses potenciais inerentes à relativa imprecisão de inquéritos alimentares e ao fato de que nosso método não testou a aplicação dos marcadores via formulário do Sisvan no serviço de saúde. Apesar de não termos conhecimento de estudos que tenham comparado os indicadores obtidos do questionário do Sisvan com métodos de coleta de dados de consumo alimentar mais abrangentes, outros trabalhos já mostraram uma boa concordância entre relatos de *screeners* do consumo alimentar e estimativas de recordatórios de 24 horas ^36 , 37^ . Além disso, análises recentes, também com objetivo de fornecerem evidências da validação dos marcadores do consumo alimentar do Sisvan, indicaram que o formulário apresenta estrutura interna bidimensional para indivíduos a partir de dois anos de idade, contrapondo os quatro itens que congregam alimentos ultraprocessados (dimensão 1) àqueles que incluem frutas, hortaliças e feijão (dimensão 2), e possui características de mensuração consistentes entre macrorregiões, faixas etárias e ao longo dos anos ^38^ .

Por outro lado, o caráter rigorosamente probabilístico da amostra estudada, e a representatividade nacional, assegurada com o estudo de mais de 40 mil pessoas residentes nas áreas urbanas e rurais das várias regiões do País, a coleta de dados de consumo alimentar feita com diversas estratégias de controle de qualidade e por meio de software validado, além da disposição de base de dados com mais de 2000 itens alimentares, são grandes fortalezas do estudo. Além disso, a consistência dos achados pode ser observada com a avaliação dos marcadores do Sisvan em comparação com diferentes indicadores amplamente reconhecidos de qualidade da dieta.

Em conclusão, este estudo demonstrou que os marcadores do consumo alimentar do Sisvan possuem bom desempenho para refletirem a qualidade global da alimentação de adolescentes e adultos brasileiros. Considerando que esses marcadores estão incorporados ao SUS e são aplicados de forma rápida e prática pelos profissionais da APS, reafirma-se seu papel central na estratégia de vigilância alimentar e nutricional do País.

## Footnotes

a Acesso disponível em: http://www.fcf.usp.br/tbca
